# A Hyperlipidic Diet Combined with Short-Term Ovariectomy Increases Adiposity and Hyperleptinemia and Decreases Cytokine Content in Mesenteric Adipose Tissue

**DOI:** 10.1155/2015/923248

**Published:** 2015-06-14

**Authors:** Nelson Inacio Pinto Neto, Maria Elizabeth Sousa Rodrigues, Ana Claudia Losinskas Hachul, Mayara Franzoi Moreno, Valter Tadeu Boldarine, Eliane Beraldi Ribeiro, Lila Missae Oyama, Claudia Maria Oller do Nascimento

**Affiliations:** Departamento de Fisiologia, Disciplina de Fisiologia da Nutrição, Universidade Federal de São Paulo, Rua Botucatu 862, 2nd floor, Edifício de Ciências Biomédicas, 04023060 Vila Clementino, SP, Brazil

## Abstract

Four-week-old female Wistar rats were divided into two groups and fed a control diet (C) or a hyperlipidic diet (H) for 4 weeks. Rats from each group underwent ovariectomy (OVX) or sham surgery (SHAM). They received C or H for the next four weeks. The body weight gain (BW), food efficiency (FE), and carcass lipid content were higher in the OVX H than in the SHAM H. The OVX H exhibited a higher serum leptin level than other groups. IL-6, TNF-*α*, and IL-10 content of mesenteric (MES) adipose tissue was lower in the OVX H than in the OVX C. IL-6, TNF-*α*, and IL-10 content of retroperitoneal (RET) adipose tissue was lower in the SHAM H than in the SHAM C. The SHAM H showed decreased TG relative to the SHAM C. Similar results were obtained in relation to IL-6R*α*, TNFR1, TLR-4, and MyD88 contents in the MES and RET white adipose tissue among the groups. A hyperlipidic diet for 8 weeks combined with short-term ovariectomy decreases the cytokine content of MES adipose tissues but increases BW, enhancing FE and elevating serum leptin levels. These suggest that the absence of estrogens promotes metabolic changes that may contribute to installation of a proinflammatory process induced by a hyperlipidic diet.

## 1. Introduction

An increasingly sedentary lifestyle combined with rising consumption of caloric foods has transformed obesity into a global event [1]. Obesity is a low-level, chronic inflammatory disease with a multifactorial etiology that includes eating habits, a sedentary lifestyle, and genetic predisposition that promotes the excessive accumulation of body fat [2, 3].

In addition to its roles as an energy reservoir and heat insulating material, adipose tissue also secretes cell-signaling molecules such as adipokines [2]. Adipose tissue secretes proinflammatory factors like interleukin 6 (IL-6), tumor necrosis factor-alpha (TNF-*α*), and leptin and anti-inflammatory factors as and interleukin 10 (IL-10) and adiponectin [4].

Studies have demonstrated that the administration of a high-fat diet in rodents promotes metabolic changes, inducing the production of proinflammatory interleukins and a chronic inflammatory process [5, 6]. The presence of large concentrations of endotoxins and saturated fatty acids in the diet promotes the activation of Toll-like receptor 4 (TLR-4), which triggers the production of proinflammatory cytokines [4, 5]. Through receptors associated with transmembrane proteins, these cytokines interfere with the expression of other cytokines, thus affecting metabolic homeostasis [7, 8].

Among various implications that are closely correlated with obesity, menopause deserves particular attention because of the increase in life expectancy of the population [9]. Menopause is defined as the permanent cessation of menstruation due to loss of ovarian function [9, 10]. The loss of ovarian function, particularly the decline in estrogen production, promotes adverse changes in the profile of lipoproteins, metabolism of glucose and insulin, distribution of body fat, clotting, and vascular endothelium. In combination with weight gain, these changes place women at a high risk of developing cardiovascular diseases, which are very common in this period of life [10, 11].

The physiological actions of estrogens in the body are mediated by two distinct estrogen receptors (ERs): ER*α* and ER*β*; these receptors are nuclear transcription factors involved in the regulation of several complex physiological processes [12, 13]. According to Benedusi and colleagues, the inflammatory processes that occur following the menopause result from a reduction in circulating levels of estrogens and their receptors (ERs), which have anti-inflammatory properties. However, controversy persists as to whether the effects of estrogens are anti- or proinflammatory. Studies in premenopausal women suggest a beneficial role for estrogen in the prevention of vascular inflammation and consequent atherosclerosis. Estrogen exerts an anti-inflammatory effect on the vasculature, through antioxidant effects, generation of nitric oxide, prevention of apoptosis in vascular cells, suppression of proinflammatory cytokines, and modulation of the renin-angiotensin system [14].* In vitro* studies have also demonstrated that estrogen has an anti-inflammatory effect in several cell lineages [15].

The hormonal changes that occur during the menopause are responsible for specific physical and metabolic remodeling that, when associated with weight gain and obesity, have a negative impact on women's health. Therefore, it is essential that both obesity and menopause, in particular the effects of estrogen on the inflammatory state, are investigated to improve women's quality of life [16]. In this study, we evaluated whether a high-fat diet with or without ovariectomy promoted the inflammatory process in rats by assessing the production of pro- and anti-inflammatory factors and their receptors and TLR-4 protein content.

## 2. Material and Methods

### 2.1. Animals and Experimental Conditions

The Experimental Research Committee of the São Paulo Federal University approved all procedures for the care of the animals used in this study. Thirty-day-old female Wistar rats were used in this study and were kept under controlled conditions of light (12 : 12 h light–dark cycle with lights on at 07:00) and temperature (22 ± 1°C). During the experimental period the animals were maintained in collective cages and received water and the specific diet ad libitum.

Thirty-day-old rats were divided into two groups and fed for 4 weeks with one of the following diets: control diet (C) or hyperlipidic diet (H).

After this period the animals were divided into four groups:

1. SHAM C: sham group treated with control diet;
2. SHAM H: sham group treated with hyperlipidic diet;
3. OVX C: ovariectomized group treated with control diet;
4. OVX H: ovariectomized group treated with hyperlipidic diet.

The food intake and body weight were measured weekly at 09:00.

The control diet was prepared according to the recommendations of the American Institute of Nutrition (AIN-93M) [17] and the hyperlipidic diet was by AIN-93M modified. The fat content in the control diet (C) was 4% containing 0.64 g of saturated fatty acids, and the hyperlipidic diet containing 20% of fat which 7.7 g was saturated fatty acids. The energy content of C diet was 4114.71 KJ and of HC diet 6438.58 KJ/100 g (Table 1).

### 2.2. Ovariectomy

At 60 days of age, the rats were anesthetized and received intraperitoneal injection of ketamine (70 mg/kg) and xylazine (10 mg/kg). The OVX group was submitted to bilateral ovariectomy. For this, they were subjected to a muscular incision to open the peritoneal cavity for posterior connection of the uterine tubules and removal of the ovaries. Afterward, the peritoneal cavity was sutured and cleaned. The female sham group only underwent an incision. All the animals received antibiotic (penicillin) immediately after the surgery and 0.1 mL/kg body weight of ibuprofen 50 mg for 2 days.

### 2.3. Experimental Procedure

The animals were euthanized by decapitation on the 90th day of life in the fasting state (10 h) in the early morning to avoid chronobiological variations. The SHAM groups were in estrus phase (phase when estrogen secretions exert their biggest influence). Trunk blood was collected and immediately centrifuged at 4°C and serum aliquots were taken and frozen at −80°C to measure the concentrations of glucose, triacylglycerols, and total cholesterol using commercials kits from Labtest Diagnostic SA (MG, Brazil). The concentrations of insulin, leptin, and adiponectin were determined by ELISA (Linco Research Inc., USA).

The retroperitoneal [7], parametrial (PAR), and mesenteric (MES) adipose tissues, gastrocnemius muscle (GAST), and liver were dissected, weighed, frozen in liquid nitrogen, and stored at −80°C until the protein extraction.

### 2.4. Carcass Lipid and Protein Content

For determination of carcass lipid and protein content, the carcasses were eviscerated, weighed, and stored at −20°C. Lipid content was measured as described by Stansbie et al. [18] and standardized using the method described by Oller do Nascimento and Williamson [19]. Briefly, the eviscerated carcass was autoclaved at 120°C for 90 min and homogenized with double the mass of water. Triplicate aliquots of this homogenate were weighed and digested in 3 mL of 30% KOH and 3 mL of ethanol for at least 2 h at 70°C in capped tubes. After cooling, 2 mL of 12 N H_2_SO_4_ was added, and the sample was washed three times with petroleum ether for lipid extraction. Results are expressed as grams of lipid/100 g of carcass. For protein measurements, aliquots of the same homogenate (approximately 1 g) were heated to 37°C for 1 h in 0.6 N KOH with constant shaking. After clarification by centrifugation, protein content was measured according to Bradford (Bio-Rad, Hercules, CA).

### 2.5. Tissues Protein Extraction and Determination

Adipose tissue depots, gastrocnemius muscle, and liver (0.15–0.3 g) were homogenized in ice-cold solubilization and total protein extraction buffer (100 mM Tris, pH 7.5, 100 mM sodium fluoride, 10 mM sodium orthovanadate, 2 mM phenylmethylsulfonyl fluoride, 10 mM sodium pyrophosphate, and 0.1 mg/mL aprotinin). After homogenization, Triton X-100 was added to a final concentration of 1%. Samples rested on ice for 30 min and were clarified by centrifugation. Homogenates were centrifuged at 19283 ×g for 40 min at 4°C. The supernatants were saved and the protein concentrations were determined using a Bradford assay (Bio-Rad, Hercules, CA) with bovine serum albumin as a reference. Quantitative assessment of IL-6, IL-10, and TNF-*α* was carried out using ELISA. The results are expressed as pg/g of protein.

### 2.6. Western Blot Analysis

Fifty micrograms of the specific tissue total protein was loaded onto a sodium dodecyl sulfate-polyacrylamide gel (5% stacking gel; 10% running gel), separated by electrophoresis, and then electroblotted onto nitrocellulose membranes (Hybond-C Extra, Amersham) using a wet electroblotter (Bio-Rad, CA, USA). After blotting, the membranes were blocked in Tris-buffered saline- (TBS-) Tween buffer, pH 7.5 (20 mM Tris/500 mM NaCl/0.05% Tween-20), containing 1% bovine serum albumin and then exposed to specific antibodies diluted in TBS-Tween buffer (pH 7.5) containing 1% BSA for 2 h. The membranes then were washed and incubated with anti-rabbit Ig or anti-mouse Ig conjugated to horseradish peroxidase and diluted to 1/1000 in the same buffer for 1 h. After a series of washes in TBS-Tween buffer, the bands were visualized with enhanced chemiluminescence scanned at UVITec (Cambridge) after adding the ECL reagent (GE Healthcare Bio-Sciences AB, UK). The size of the protein bands was determined using electrophoresis color markers. The antibodies against anti-TLR4 (sc-99183), anti-TNF-R1 (sc-7895), anti-IL-6 R*α* (sc-660), anti-MyD88 (sc-8197), and anti-*α*-tubulin (sc-58667) were obtained from Santa Cruz Biotechnology (Santa Cruz, CA, USA), and the anti-rabbit Ig and anti-mouse Ig conjugated to horseradish peroxidase were obtained from Sigma (USA). A quantitative analysis of the blots was performed using ImageJ software.

### 2.7. Statistical Analysis

The data are presented as mean ± SEM. The results were analyzed using two-way analysis of variance followed by Tukey's test. Differences were considered significant when *P* < 0.05.

## 3. Results

### 3.1. Body and Tissue Weight and Carcass Protein and Lipid Content

The initial body, liver, GAST, and PAR weights were similar in all groups. In the OVX H group, the RET weight was higher than in the SHAM C group and the MES weight was higher than in the SHAM H group. Administration of a hyperlipidic diet in the SHAM group did not modify these parameters compared with the administration of a control diet. However, the association of a hyperlipidic diet with ovariectomy (OVX H) caused a significant increase in final body weight. Carcass protein content was greater in the OVX C group than both SHAM groups. However, carcass lipid content in the OVX H group was higher than that in the SHAM H group (Table 2).

### 3.2. Body Weight Gain and Food Efficiency

**Figure 1:**
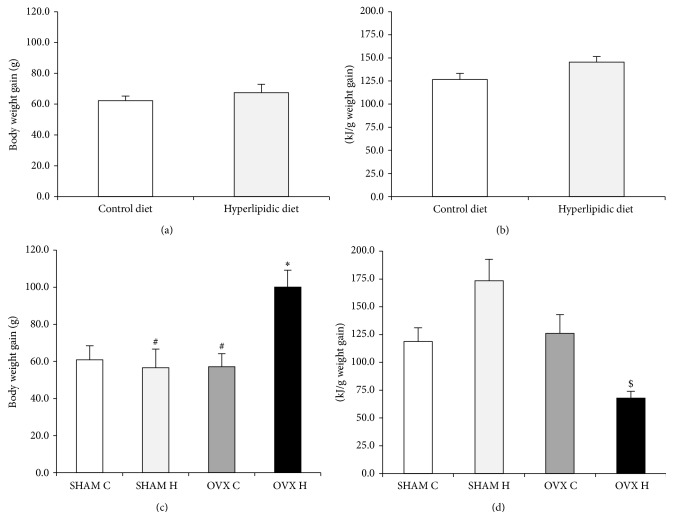
Body weight gain after four weeks of control diet (number of animals is 15) or hyperlipidic diet (number of animals is 13) (a) and after four weeks of ovariectomy (b) of studied rats groups: (SHAM C) sham group treated with control diet; (SHAM H) sham group treated with hyperlipidic diet; (OVX C) ovariectomized group treated with control diet; (OVX H) ovariectomized group treated with hyperlipidic diet. The numbers of animals varied between six and eight. ^∗^ *P* < 0.05 as compared to the SHAM C; ^#^ *P* < 0.05 as compared to OVX H; ^$^ *P* < 0.05 as compared to SHAM H.

The initial 4 weeks of hyperlipidic diet administration did not modify body weight gain and food efficiency (Figures 1(a) and 1(b)). However, the combination of ovariectomy and administration of a hyperlipidic diet (OVX H) increased body weight gain accompanied by high food efficiency (Figures 1(c) and 1(d)).

### 3.3. Serum Lipid Profile and Glucose, Adiponectin, Leptin, and Insulin Concentrations

The serum concentrations of total cholesterol, HDL (high-density lipoprotein) cholesterol, glucose, adiponectin, and estradiol were similar in all groups. Serum levels of triacylglycerol (TG) were decreased in the SHAM H group compared with the SHAM C group. Serum insulin concentration was lower in the SHAM H, OVX C, and OVX H groups than in the SHAM C group. Ovariectomy combined with the administration of a hyperlipidic diet caused an increase in serum leptin levels compared with all other groups (Table 3).

### 3.4. Tissues Cytokine Content

**Figure 2:**
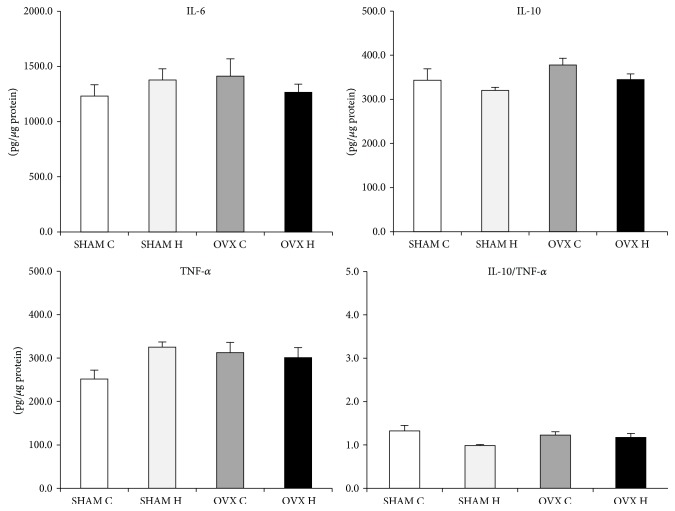
Liver cytokines content of studied rats groups: (SHAM C) sham group treated with control diet; (SHAM H) sham group treated with hyperlipidic diet; (OVX C) ovariectomized group treated with control diet; (OVX H) ovariectomized group treated with hyperlipidic diet. The numbers of samples varied between six and eight.

**Figure 3:**
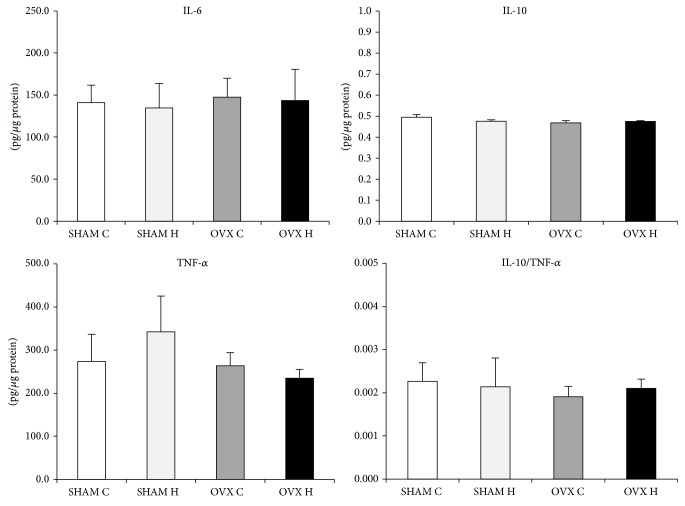
Gastrocnemius muscle cytokines content of studied rats groups: (SHAM C) sham group treated with control diet; (SHAM H) sham group treated with hyperlipidic diet; (OVX C) ovariectomized group treated with control diet; (OVX H) ovariectomized group treated with hyperlipidic diet.

**Figure 4:**
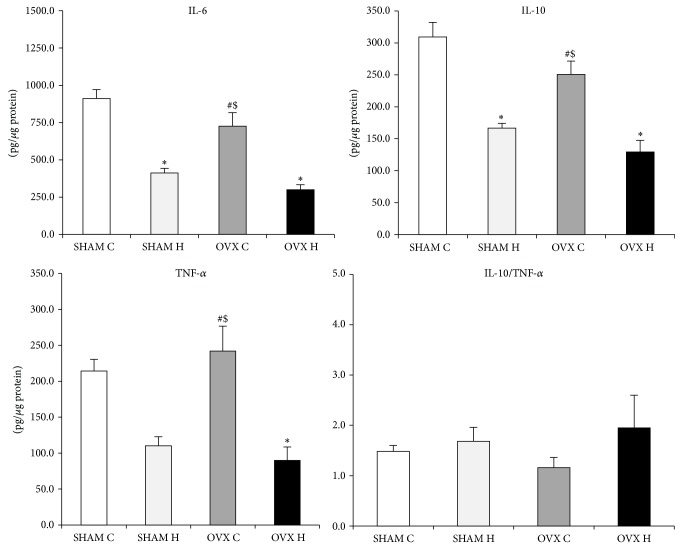
Mesenteric adipose tissue cytokines content of studied rats groups: (SHAM C) sham group treated with control diet; (SHAM H) sham group treated with hyperlipidic diet; (OVX C) ovariectomized group treated with control diet; (OVX H) ovariectomized group treated with hyperlipidic diet. The numbers of samples varied between six and eight. ^∗^ *P* < 0.05 as compared to the SHAM C; ^#^ *P* < 0.05 as compared to OVX H; ^$^ *P* < 0.05 as compared to SHAM H.

The cytokines content was similar in the SHAM C, SHAM H, OVX C, and OVX H groups and in the liver and GAST (Figures 2 and 3). However, administration of a hyperlipidic diet caused a decrease in IL-6 and IL-10 levels in MES adipose tissues. Furthermore, administration of a hyperlipidic diet associated with ovariectomy decreased the TNF-*α* content of MES (Figure 4).

**Figure 5:**
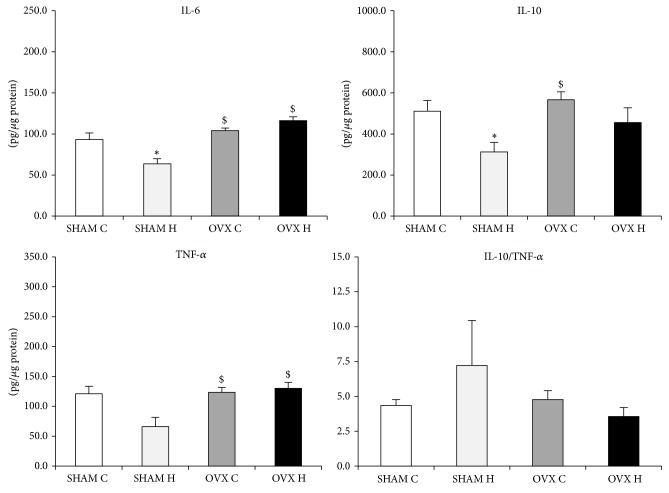
Retroperitoneal adipose tissue cytokines content of studied rats groups: (SHAM C) sham group treated with control diet; (SHAM H) sham group treated with hyperlipidic diet; (OVX C) ovariectomized group treated with control diet; (OVX H) ovariectomized group treated with hyperlipidic diet. The numbers of samples varied between six and eight. ^∗^ *P* < 0.05 as compared to the SHAM C; ^#^ *P* < 0.05 as compared to OVX H; ^$^ *P* < 0.05 as compared to SHAM H.

The IL-6 content was decreased by the hyperlipidic diet; however, in combination with ovariectomy, this effect was reversed in RET adipose tissues (Figure 5). The hyperlipidic diet caused a decrease in IL-10 levels, but ovariectomy had the opposite effect, and the TNF-*α* content of RET adipose tissues was high following ovariectomy (Figure 5).

### 3.5. IL-6 Receptor *α* (IL-6R*α*), TNF Receptor 1 (TNFR1), TLR-4, and Myeloid Differentiation Primary Response (MyD88) Protein Content in the Studied Tissues

**Figure 6:**
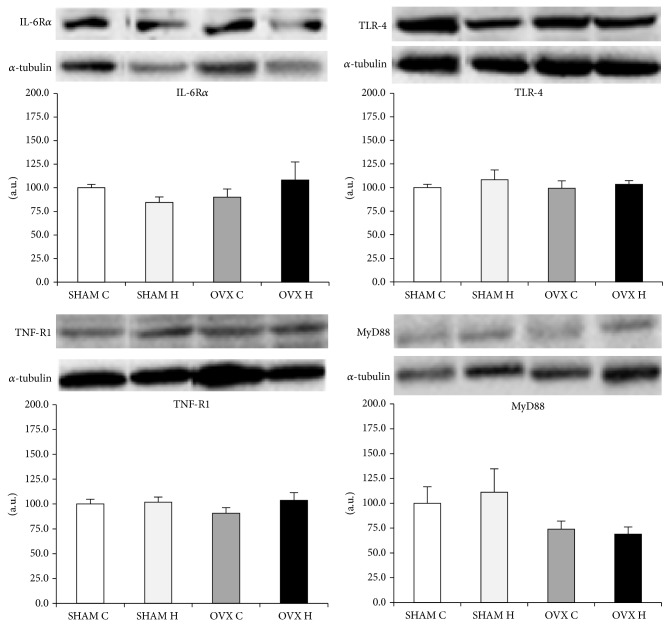
Liver IL-6R*α*, TNF-R1, TLR-4, and MyD88 protein content: (SHAM C) sham group treated with control diet; (SHAM H) sham group treated with hyperlipidic diet; (OVX C) ovariectomized group treated with control diet; (OVX H) ovariectomized group treated with hyperlipidic diet. The numbers of samples varied between four and six.

**Figure 7:**
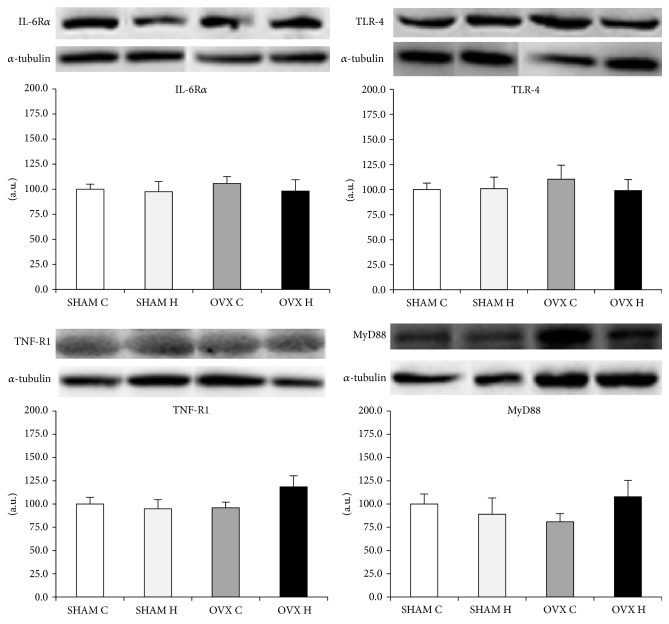
Gastrocnemius muscle IL-6R*α*, TNF-R1, TLR-4, and MyD88 protein content: (SHAM C) sham group treated with control diet; (SHAM H) sham group treated with hyperlipidic diet; (OVX C) ovariectomized group treated with control diet; (OVX H) ovariectomized group treated with hyperlipidic diet.

**Figure 8:**
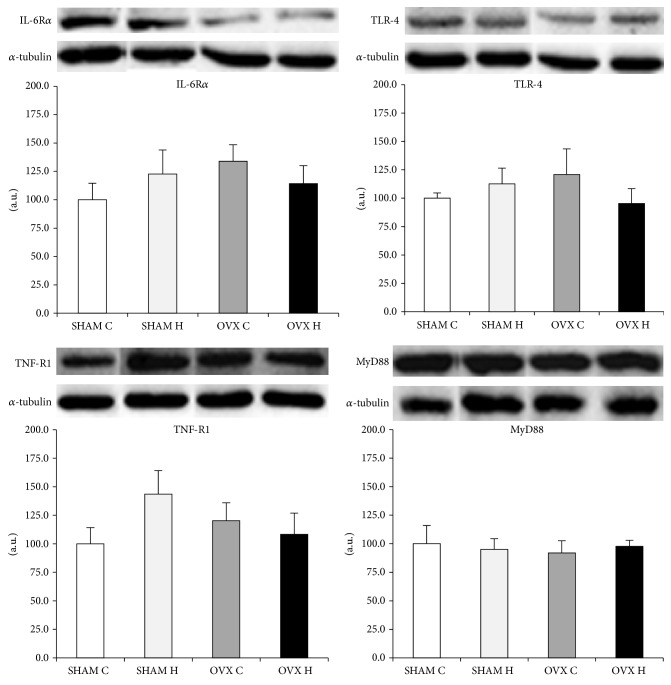
Mesenteric adipose tissue IL-6R*α*, TNF-R1, TLR-4, and MyD88 protein content: (SHAM C) sham group treated with control diet; (SHAM H) sham group treated with hyperlipidic diet; (OVX C) ovariectomized group treated with control diet; (OVX H) ovariectomized group treated with hyperlipidic diet.

The IL-6 receptor, TNF receptor, TLR-4, and MyD88 protein content did not differ between groups in the liver (Figure 6), GAST (Figure 7), and MES adipose tissues (Figure 8).

## 4. Discussion

In this study, we demonstrated that administration of a hyperlipidic diet for 8 weeks combined with short-term ovariectomy promoted an increase in body weight gain and a decrease in metabolic efficiency, accompanied by hyperleptinemia. The cytokines content of the liver and GAST was unchanged; however, levels of all cytokines analyzed were decreased in MES adipose tissues. Also, we demonstrated that 4 weeks of ovariectomy (OVX C group)* per se* did not modify these parameters compared with the SHAM C group.

However, Ryou et al. [20] demonstrated that 8 weeks of ovariectomy in rats caused an increase in body weight and food efficiency with elevated plasma leptin. These results suggest that the time period after ovariectomy may influence body weight gain and food efficiency.

Previously, Jen et al. [21] showed that high-fat diet fed male rats were heavier than chow-fed male rats by the sixth week of a high-fat diet, whereas female rats were heavier by the ninth week. Consistent with this, we demonstrated that the administration of a hyperlipidic diet for 8 weeks compared with a control diet did not cause an increase in body weight gain in female rats. Gorres et al. [22] showed that ingestion of a high-fat diet for 8 weeks did not modify body or tissue weight. However, the combination of a high-fat diet and ovariectomy caused an increase in body weight gain accompanied by an increase in RET and MES weight, similar to our findings.

This excessive weight gain is primarily due to fat deposition, reflected by increased serum leptin levels, which rise in direct proportion to fat mass [23]. Carcass lipid content in the OVX H group was higher than in the SHAM H and SHAM C groups. Diets rich in saturated fatty acids decrease uncoupling protein-1 (UCP-1) activity in brown adipose tissue [24]. However, controversy surrounds the effect of ovariectomy on energy expenditure. Liu et al. [25] showed that low estrogens levels, characteristic of ovariectomy, increase UCP-1 expression in subcutaneous adipose tissue from 4 weeks until 12 weeks of ovariectomy. In contrast, Ko et al. [26] indicated that a high-fat diet combined with ovariectomy decreased UCP-1 levels relative to controls.

Also, it has been reported that ovariectomy causes a decrease in the lipolytic responsiveness to norepinephrine of fat cells and an increase in adipose tissue lipoprotein lipase (LPL) [27]. LPL hydrolyzes TG from chylomicrons and very-low-density lipoproteins to free fatty acids and monoacylglycerols. These products are reesterified and stored as TG in fat cells. A hyperlipidic diet was shown to cause an increase in LPL activity in white adipose tissue [28]. By these mechanisms, ovariectomy promotes increased adiposity, particularly in rats receiving a hyperlipidic diet.

Taken together, these findings imply that a high-fat diet combined with ovariectomy may cause a decrease in energy expenditure and an increase in adipose tissue deposits, probably via enhanced uptake of TG from chylomicrons. Also, in the present study, it was observed that serum TG was lower in SHAM H group compared with SHAM C group. Studies have reported that low-fat, high-carbohydrate diets increase the liver “*de novo*” lipogenesis and cause hypertriglyceridemia compared to high-fat diets [29, 30].

Amengual-Cladera et al. [31] showed that the administration of estrogen promoted a slight decrease in body weight without entailing a significant reduction in adiposity index or in RET weight in obese rats. These data suggest that this treatment is insufficient to restore these parameters, although there is a 77% reduction in the expression of LPL, which would reduce fat deposition by decreasing fatty acid uptake from the circulation. However, this is inconsistent with studies in which estrogen replacement managed to recover both body weight and adiposity index in female control rats. This discrepancy may be related to the structural form of estrogen used, in addition to the dosage and duration of estrogen replacement and the age at which the female rats were ovariectomized. These notes can justify the data found in our study.

Increase in serum leptin concentration was observed only in OVX H group, suggesting that the decrease in estrogen could potentiate the effect of high-fat diet on leptin secretion. Ludgero-Correia et al. [32] observed a similar result. Hyperleptinemia may arise as a result of the increased body weight of rats in the OVX H group. Saravanan et al. [33] showed that animals fed a high-fat diet were hyperleptinemic and overweight compared with animals receiving a control diet. It is established that serum leptin levels rise with increasing adiposity and are directly proportional to fat mass. Loss of efficacy of endogenous or exogenous leptin in the therapeutic maintenance of body weight is attributable to the development of resistance to leptin, a common occurrence in obese individuals. Possible explanations for this include a lack of leptin at central sites resulting from defective transport, for instance, through the blood and the brain barrier, decreased production of leptin by the hypothalamus, or interruption of signal transduction between leptin and receptors in the hypothalamus [24].

Saravanan et al. [33] showed that animals fed a high-fat diet had a state of hyperleptinemia accompanied by overweight when compared to animals that received control diet.

It is understood that ovariectomy promotes hyperphagia and weight gain, and although this hyperphagia is transitional, the gain in body weight is more durable. Estrogen replacement has been shown to decrease food intake and restore weight in ovariectomized rats. These facts suggest that estrogen is involved in the modulation of energy homeostasis by direct action on the appetite and central pathways regulating energy and by controlling the secretion of hormonal signals peripherals such as leptin, which in turn regulates effector pathways central and essential to the integration of the hypothalamus in energy homeostasis [34].

Inflammatory markers are directly related to inflammation in obesity. In relation to cytokines content, administration of a hyperlipidic diet in this study caused a decrease in IL-6 and IL-10 content in both RET and MES adipose tissues. Previously, our group [35] showed that administration of a lard diet for 8 weeks promoted a decrease in IL-10 in RET and MES adipose tissues of male mice, with no effect on IL-6 or TNF-*α*. Cani et al. [36] observed an increase in LPS, accompanied by elevated mRNA concentrations of PAI-1, IL-1, TNF-*α*, and F4/80 in subcutaneous adipose depots and PAI-1, IL-1, and F4/80 in MES adipose tissues, of high-fat diet fed male mice relative to control diet fed mice. These results suggest that the presence of female hormones could modify the response to a high-fat diet in terms of IL-6 adipose tissue content, especially in RET adipose tissues.

Previously, Lafontan and Berlan [37] reported that the physiology, metabolism, and function of white adipose tissue vary in a depot-specific manner. Several factors could contribute to this, such as difference in the amount of hormones receptors, blood flow, hormone, cytokines, and polypeptides production. For instance, it has been demonstrated by Yamashita et al. [38] that aerobic training was effective in reducing adipokines levels related to inflammation in mesenteric adipose tissue but not in retroperitoneal adipose tissue.

In this study, the rats were euthanized, between 9:00 and 12:00 h, in the estrus phase, a period when estrogen secretions exert their biggest influence. In fact,* in vitro* studies demonstrated an inhibitory effect of estradiol on IL-6 production in several cell types in humans and rodents, including macrophages, monocytes, osteoblasts, and bone stromal cells [39–41].

Conversely, ovariectomy combined with a hyperlipidic diet promoted a decrease in the TNF-*α*, IL-6, and IL-10 content of MES adipose tissues. Riant et al. [42] demonstrated that estrogens, particularly chronic E2 administration to ovariectomized mice, enhance the expression of inflammatory factors, such as IL-6 and TNF-*α*, in mice fed with a high-fat diet for 4 or 12 weeks, indicative of a proinflammatory effect of E2, at least in visceral adipose tissues. Accordingly, in this study, we observed a decrease in TNF-*α* in MES adipose tissues in the OVX H group compared with the SHAM H group, supporting the idea of a possible* in vivo* proinflammatory effect of estrogen on white adipose tissue, in special visceral one.

Both hyperlipidic diet and estrogen have been reported to influence the circadian rhythm modifying the expression of clock genes involved in the regulation of metabolism and protein expression [43, 44]. In this sense, Cano et al. [45] demonstrated that high-fat diet disrupts the daily variations of circulating of several adipocytokines, particularly by a reduction in plasma concentration of TNF-*α* from 9:00 to 13:00 h, compared to control diet treated rats.

It could be speculated that the results found in the present study, related to the effect of diet and ovariectomy on adipose tissues cytokines content, could be related to the disruption on the clock genes, since the presence of circadian clock genes has been reported in fat [46]. Further studies are needed to confirm this hypothesis.

Despite the modification of cytokines content in white adipose tissue by the administration of a hyperlipidic diet and/or ovariectomy, protein levels of IL-6R*α*, TNFR1, TLR-4, and MyD88 were similar between groups in all tissues studied (Figures 6, 7, and 8). Activation of the TLR-4 signaling pathway involves both MyD88-dependent and MyD88-independent signaling [47]. MyD88 knockout mice showed no response to the TLR-4 ligand LPS in terms of inflammatory mediator production by macrophages, B cell proliferation, or endotoxin shock [47, 48]. However, in the case of TLR-4 stimulation, LPS-induced activation of NF-*κ*B and c-Jun N-terminal kinases (JNK) was observed with delayed kinetics, even in MyD88 knockout cells, although these cells did not produce any inflammatory cytokines in response to LPS [48]. Taken together, it is possible to speculate that the modification in cytokines content in white adipose tissue observed in this study may have been stimulated by another mechanism, such as MAPK dependent pathway.

In conclusion, our results demonstrate that administration of a hyperlipidic diet for 8 weeks combined with short-term ovariectomy did not alter the cytokine content of the liver and gastrocnemius muscle but caused a decrease in all analyzed cytokines in mesenteric adipose tissue. However, ovariectomy in rats fed a hyperlipidic diet caused hyperleptinemia and increased body weight gain, food efficiency, and carcass lipid content, which, in combination with long-term ovariectomy, may contribute to the induction of proinflammatory processes, particularly those associated with a high-fat diet. These results emphasize that, after the menopause, women must decrease their consumption of fat.

## Figures and Tables

**Table 1:** Composition of the control (C) hyperlipidic (H) diets prepared according to AIN-93.

Ingredient	Diet (g/1000 g)	
	C	H
Cornstarch	720.7	500.0
Soybean oil	40.0	20.0
Lard	—	180.0
Casein	140.0	151.0
L-Cystine	1.8	1.8
Cellulose	50	—
Saccharose	—	100
Mineral mixture	35.0	35.0
Vitamin mixture	10.0	10.0
Bitartrate choline	2.5	2.5
Butylhydroquinone	0.008	0.008

**Table 2:** Body weight, tissue weight, sum of adipose tissue depots, carcass protein, and lipid content of studied rats groups.

	SHAM C	SHAM H	OVX C	OVX H
Body weight (g)				
Initial	116.39 ± 4.32	116.24 ± 3.24	106.71 ± 4.24	114.99 ± 6.06
Final	239.58 ± 7.33	247.70 ± 11.43	232.77 ± 7.49^#^	279.26 ± 9.82^∗^
Carcass protein content (g/100 g)	18.45 ± 1.07	17.94 ± 1.24	22.81 ± 0.95^$∗^	21.76 ± 0.95
Carcass lipid content (g/100 g)	13.18 ± 0.97	12.04 ± 0.67^#^	13.55 ± 1.40	18.08 ± 1.91
RET adipose tissues (g/100 g)	1.00 ± 0.09	1.19 ± 0.12	1.21 ± 0.17	1.59 ± 0.17^∗^
PAR adipose tissues (g/100 g)	1.28 ± 0.15	1.35 ± 0.13	0.90 ± 0.18	1.25 ± 0.12
MES adipose tissues (g/100 g)	1.04 ± 0.10	0.79 ± 0.10	1.15 ± 0.19	1.49 ± 0.17^$^
Sum of adipose tissue depots	3.31 ± 0.24	3.34 ± 0.23	3.28 ± 0.46	4.30 ± 0.31
Liver	2.35 ± 0.26	2.50 ± 0.08	2.66 ± 0.12	2.97 ± 0.14
GAST	0.99 ± 0.05	0.83 ± 0.04	0.93 ± 0.05	0.93 ± 0.04

The numbers of samples varied between six and eight. ^∗^ *P* < 0.05 as compared to the SHAM C; ^#^ *P* < 0.05 as compared to OVX H; ^$^ *P* < 0.05 as compared to SHAM H.

**Table 3:** Serum triacylglycerol, total cholesterol, HDL-cholesterol, glucose, insulin, leptin, and adiponectin of studied rats groups.

	SHAM C	SHAM H	OVX C	OVX H
Triacylglycerol (mg/dL)	157.81 ± 14.29	111.51 ± 6.94^∗^	138.57 ± 21.96	122.23 ± 11.05
Total cholesterol (mg/dL)	131.25 ± 28.75	108.08 ± 15.89	108.64 ± 12.22	133.93 ± 16.99
HDL (mg/dL)	56.02 ± 5.93	58.06 ± 3.03	62.51 ± 1.60	67.50 ± 2.99
Glucose (mg/dL)	116.54 ± 6.24	97.80 ± 1.55	106.68 ± 6.77	101.33 ± 1.42
Insulin (*µ*g/*µ*L)	1.66 ± 0.20	0.75 ± 0.13^∗^	0.99 ± 0.12^∗^	1.12 ± 0.10^∗^
Leptin (*µ*g/mL)	5.37 ± 0.58	4.98 ± 0.68^#^	4.76 ± 0.82^#^	10.10 ± 1.08^∗^
Adiponectin (*µ*g/mL)	38.33 ± 6.61	42.44 ± 1.64	48.65 ± 0.65	45.70 ± 2.25

The numbers of samples varied between six and eight. ^∗^ *P* < 0.05 as compared to the SHAM C; ^#^ *P* < 0.05 as compared to OVX H.

## References

[1] Ahmed H. G., Ginawi I. A., Elasbali A. M., Ashankyty I. M., Al-Hazimi A. M. (2014). Prevalence of obesity in Hail region, KSA: in a comprehensive survey. *Journal of Obesity*.

[2] Makki K., Froguel P., Wolowczuk I. (2013). Adipose tissue in obesity-related inflammation and insulin resistance: cells, cytokines, and chemokines. *ISRN Inflammation*.

[3] Sakurai T., Ogasawara J., Kizaki T. (2013). The effects of exercise training on obesity-induced dysregulated expression of adipokines in white adipose tissue. *International Journal of Endocrinology*.

[4] Lee H., Lee I. S., Choue R. (2013). Obesity, inflammation and diet. *Pediatric Gastroenterology, Hepatology & Nutrition*.

[5] Enos R. T., Velázquez K. T., Murphy E. A. (2014). Insight into the impact of dietary saturated fat on tissue-specific cellular processes underlying obesity-related diseases. *The Journal of Nutritional Biochemistry*.

[6] Sarvas J. L., Niccoli S., Walser E., Khaper N., Lees S. J. (2014). Interleukin-6 deficiency causes tissue-specific changes in signaling pathways in response to high-fat diet and physical activity. *Physiological Reports*.

[7] Derouet D., Rousseau F., Alfonsi F. (2004). Neuropoietin, a new IL-6-related cytokine signaling through the ciliary neurotrophic factor receptor. *Proceedings of the National Academy of Sciences of the United States of America*.

[8] Pedersen B. K., Febbraio M. A. (1985). Point: interleukin-6 does have a beneficial role in insulin sensitivity and glucose homeostasis. *Journal of Applied Physiology*.

[9] Lee J. O., Kang S. G., Kim S. H., Park S. J., Song S. W. (2011). The relationship between menopausal symptoms and heart rate variability in middle aged women. *Korean Journal of Family Medicine*.

[10] Kilim S. R., Chandala S. R. (2013). A comparative study of lipid profile and oestradiol in pre- and post-menopausal women. *Journal of Clinical and Diagnostic Research*.

[11] Ga W. (2012). Estado nutricional e qualidade de vida da mulher climatérica. *Revista Brasileira de Ginecologia e Obstetrícia*.

[12] Weihua Z., Saji S., Makinen S. (2000). Estrogen receptor (ER) beta, a modulator of ERalpha in the uterus. *Proceedings of the National Academy of Sciences of the United States of America*.

[13] Benedusi V., Meda C., Della Torre S., Monteleone G., Vegeto E., Maggi A. (2012). A lack of ovarian function increases neuroinflammation in aged mice. *Endocrinology*.

[14] Fan G.-W., Zhang Y., Jiang X. (2013). Anti-inflammatory activity of baicalein in LPS-stimulated RAW264.7 macrophages via estrogen receptor and NF-*κ*B-dependent pathways. *Inflammation*.

[15] Soucy G., Boivin G., Labrie F., Rivest S. (2005). Estradiol is required for a proper immune response to bacterial and viral pathogens in the female brain. *Journal of Immunology*.

[16] Macciò A., Madeddu C. (2011). Obesity, inflammation, and postmenopausal breast cancer: therapeutic implications. *TheScientificWorldJOURNAL*.

[17] Reeves P. G., Nielsen F. H., Fahey G. C. (1993). AIN-93 purified diets for laboratory rodents: final report of the American Institute of Nutrition ad hoc writing committee on the reformulation of the AIN-76A rodent diet. *Journal of Nutrition*.

[18] Stansbie D., Denton R. M., Bridges B. J., Pask H. T., Randle P. J. (1976). Regulation of pyruvate dehydrogenase and pyruvate dehydrogenase phosphate phosphatase activity in rat epididymal fat pads. Effects of starvation, alloxan diabetes and high fat diet. *Biochemical Journal*.

[19] Oller do Nascimento C. M., Williamson D. H. (1986). Evidence for conservation of dietary lipid in the rat during lactation and the immediate period after removal of the litter. Decreased oxidation of oral [1-14C]triolein. *Biochemical Journal*.

[20] Ryou S. H., Kang M. S., Kim K. I., Kang Y. H., Kang J. S. (2012). Effects of green tea or *Sasa quelpaertensis* bamboo leaves on plasma and liver lipids, erythrocyte Na efflux, and platelet aggregation in ovariectomized rats. *Nutrition Research and Practice*.

[21] Jen K.-L. C., Greenwood M. R. C., Brasel J. A. (1981). Sex differences in the effects of high-fat feeding on behavior and carcass composition. *Physiology and Behavior*.

[22] Gorres B. K., Bomhoff G. L., Gupte A. A., Geiger P. C. (2011). Altered estrogen receptor expression in skeletal muscle and adipose tissue of female rats fed a high-fat diet. *Journal of Applied Physiology*.

[23] Frederich R. C., Hamann A., Anderson S., Lollmann B., Lowell B. B., Flier J. S. (1995). Leptin levels reflect body lipid content in mice: evidence for diet-induced resistance to leptin action. *Nature Medicine*.

[24] Dube M. G., Beretta E., Dhillon H., Ueno N., Kalra P. S., Kalra S. P. (2002). Central leptin gene therapy blocks high-fat diet-induced weight gain, hyperleptinemia, and hyperinsulinemia: increase in serum ghrelin levels. *Diabetes*.

[25] Liu W.-H., Lee Y.-M., Lam K.-K. (2011). The role of receptor-interacting protein 140 in the accumulation of fat in ovariectomised rats. *Obesity Surgery*.

[26] Ko B.-S., Kim D. S., Kang S., Ryuk J. A., Park S. (2013). *Prunus mume* and *Lithospermum erythrorhizon* extracts synergistically prevent visceral adiposity by improving energy metabolism through potentiating hypothalamic leptin and insulin signalling in ovariectomized rats. *Evidence-Based Complementary and Alternative Medicine*.

[27] Yamaguchi M., Katoh S., Morimoto C. (2002). The hormonal responses of lipoprotein lipase activity and lipolysis in adipose tissue differ depending on the stage of the estrous cycle in female rats. *International Journal of Obesity*.

[28] Deshaies Y., Arnold J., Lalonde J., Richard D. (1988). Lipoprotein lipase in white and brown adipose tissues of exercised rats fed a high-fat diet. *The American Journal of Physiology*.

[29] Ameer F., Scandiuzzi L., Hasnain S., Kalbacher H., Zaidi N. (2014). De novo lipogenesis in health and disease. *Metabolism: Clinical and Experimental*.

[30] Schwarz J.-M., Linfoot P., Dare D., Aghajanian K. (2003). Hepatic de novo lipogenesis in normoinsulinemic and hyperinsulinemic subjects consuming high-fat, low-carbohydrate and low-fat, high-carbohydrate isoenergetic diets. *The American Journal of Clinical Nutrition*.

[31] Amengual-Cladera E., Lladó I., Gianotti M., Proenza A. M. (2012). Retroperitoneal white adipose tissue mitochondrial function and adiponectin expression in response to ovariectomy and 17*β*-estradiol replacement. *Steroids*.

[32] Ludgero-Correia A., Aguila M. B., Mandarim-de-Lacerda C. A., Faria T. S. (2012). Effects of high-fat diet on plasma lipids, adiposity, and inflammatory markers in ovariectomized C57BL/6 mice. *Nutrition*.

[33] Saravanan G., Ponmurugan P., Deepa M. A., Senthilkumar B. (2014). Anti-obesity action of gingerol: effect on lipid profile, insulin, leptin, amylase and lipase in male obese rats induced by a high-fat diet. *Journal of the Science of Food and Agriculture*.

[34] Torto R., Boghossian S., Dube M. G., Kalra P. S., Kalra S. P. (2006). Central leptin gene therapy blocks ovariectomy-induced adiposity. *Obesity*.

[35] dos Santos B., Estadella D., Hachul A. C. L. (2013). Effects of a diet enriched with polyunsaturated, saturated, or trans fatty acids on cytokine content in the liver, white adipose tissue, and skeletal muscle of adult mice. *Mediators of Inflammation*.

[36] Cani P. D., Bibiloni R., Knauf C. (2008). Changes in gut microbiota control metabolic endotoxemia-induced inflammation in high-fat diet-induced obesity and diabetes in mice. *Diabetes*.

[37] Lafontan M., Berlan M. (2003). Do regional differences in adipocyte biology provide new pathophysiological insights?. *Trends in Pharmacological Sciences*.

[38] Yamashita A. S., Lira F. S., Rosa J. C. (2010). Depot-specific modulation of adipokine levels in rat adipose tissue by diet-induced obesity: the effect of aerobic training and energy restriction. *Cytokine*.

[39] Girasole G., Jilka R. L., Passeri G. (1992). 17*β*-estradiol inhibits interleukin-6 production by bone marrow-derived stromal cells and osteoblasts in vitro: a potential mechanism for the antiosteoporotic effect of estrogens. *The Journal of Clinical Investigation*.

[40] Rogers A., Eastell R. (2001). The effect of 17*β*-estradiol on production of cytokines in cultures of peripheral blood. *Bone*.

[41] Straub R. H. (2007). The complex role of estrogens in inflammation. *Endocrine Reviews*.

[42] Riant E., Waget A., Cogo H., Arnal J.-F., Burcelin R., Gourdy P. (2009). Estrogens protect against high-fat diet-induced insulin resistance and glucose intolerance in mice. *Endocrinology*.

[43] Mendoza J., Pévet P., Challet E. (2008). High-fat feeding alters the clock synchronization to light. *The Journal of Physiology*.

[44] Zhu L., Zou F., Yang Y. (2015). Estrogens prevent metabolic dysfunctions induced by circadian disruptions in female mice. *Endocrinology*.

[45] Cano P., Cardinali D. P., Ríos-Lugo M. J., Fernández-Mateos M. P., Toso C. F. R., Esquifino A. I. (2009). Effect of a high-fat diet on 24-hour pattern of circulating adipocytokines in rats. *Obesity*.

[46] Zvonic S., Ptitsyn A. A., Conrad S. A. (2006). Characterization of peripheral circadian clocks in adipose tissues. *Diabetes*.

[47] Akira S., Takeda K., Kaisho T. (2001). Toll-like receptors: critical proteins linking innate and acquired immunity. *Nature Immunology*.

[48] Kawai T., Adachi O., Ogawa T., Takeda K., Akira S. (1999). Unresponsiveness of MyD88-deficient mice to endotoxin. *Immunity*.

